# *Rhodobacter sphaeroides* mutants overexpressing chlorophyllide *a* oxidoreductase of *Blastochloris* *viridis* elucidate functions of enzymes in late bacteriochlorophyll biosynthetic pathways

**DOI:** 10.1038/srep09741

**Published:** 2015-05-15

**Authors:** Yusuke Tsukatani, Jiro Harada, Jiro Nomata, Haruki Yamamoto, Yuichi Fujita, Tadashi Mizoguchi, Hitoshi Tamiaki

**Affiliations:** 1 Graduate School of Life Sciences, Ritsumeikan University, Shiga 525-8577, Japan; 2 PRESTO, Japan Science and Technology Agency, Saitama 332-0012, Japan; 3 Earth-Life Science Institute, Tokyo Institute of Technology, Tokyo 152-8550, Japan; 4 Department of Medical Biochemistry, Kurume University School of Medicine, Fukuoka 830-0011, Japan; 5 Graduate School of Bioagricultural Sciences, Nagoya University, Nagoya 464-8601, Japan

## Abstract

In previous studies we have demonstrated that chlorophyllide *a* oxidoreductases (CORs) from bacteriochlorophyll (BChl) *a*-producing *Rhodobacter* species and BChl *b*-producing *Blastochloris viridis* show distinct substrate recognition and different catalytic hydrogenation reactions, and that these two types of CORs therefore cause committed steps for BChls *a* and *b* biosynthesis. In this study, COR genes from *B. viridis* were incorporated and overexpressed in a series of *Rhodobacter sphaeroides* mutants. We found that the following two factors are essential in making *R. sphaeroides* produce BChl *b*: the loss of functions of both intrinsic COR and 8-vinyl reductase (BciA) in the host *R. sphaeroides* strain; and expression of the BchYZ catalytic components of COR from *B. viridis*, not the complete set of COR (BchXYZ), in the host strain. In addition, we incorporated *bchYZ* of *B. viridis* into the *R. sphaeroides* mutant lacking BchJ and BciA, resulting in the strain accumulating both BChl *a* and BChl *b*. This is the first example of an anoxygenic photosynthetic bacterium producing BChls *a* and *b* together. The results suggest that BchJ enhances activity of the intrinsic COR. The physiological significance of BchJ in pigment biosynthetic pathways will be discussed.

Chlorophyllous pigments are essential for photosynthetic organisms to harvest light energy and drive photochemical reaction centers (RCs). Phototrophic species in the phylum *Proteobacteria* (so-called purple bacteria) produce either bacteriochlorophyll (BChl) *a* or BChl *b*, depending on species, and utilize these pigments for photochemistry^1^,^2^. The difference in chemical structures between BChls *a* and *b* occurs at the C8 position, an ethyl group on BChl *a* and an ethylidene group on BChl *b* (Fig. 1). The characteristic C8-ethylidene group on BChl *b* provides the extension of the π-conjugated system on the parental bacteriochlorin ring, and gives rise to the red shift in the longest wavelength absorption band (Q_y_ band) of BChl *b* with respect to that of BChl *a*. Consequently, BChl *b* is the sole natural pigment that can efficiently absorb light energy of near-infrared wavelength (≥ 800 nm) in the monomeric state, although BChl *a* has the Q_y_ band slightly overlapping the near-infrared light region. The pigment is useful for developing artificial light-harvesting systems, such as dye-sensitized solar cells and photodynamic therapy, which are in demand to utilize uncaptured photons in longer wavelength.

Purple bacteria capture sunlight energy by light-harvesting proteins, so-called LH1 and LH2 complexes (some species have only LH1 complexes), and transfer the light energy into the type-II RC, where conversion of light energy into chemical potential energy occurs^3^. The RC and LH1 complexes bind BChl *a* or BChl *b*, depending on species, and form a supercomplex in the cytoplasmic membrane. When BChl *a* (λ_max_ = 770 nm in monomer) is incorporated into LH1 proteins, an absorption band of LH1 holoproteins usually occurs at < 900 nm in *Rhodobacter* species^4^. On the other hand, LH1 complexes binding BChl *b* (λ_max_ = 795 nm in monomer) show a significantly red-shifted absorption band at > 1000 nm in *Blastochloris viridis*^5^. Although the difference in λ_max_ wavelength in the monomeric state is 25 nm between BChls *a* and *b*, the difference between Q_y_ bands of LH1 complexes binding these two pigments is 140 nm.

Model organisms in the genus *Rhodobacter* (e.g., *R. capsulatus* and *R. sphaeroides*) are genetically amenable, have versatile ways of growing, and produce BChl pigments even when grown under dark microoxic conditions. Biosynthetic pathways for BChl *a* are well established in the *Rhodobacter* species^6^,^7^. On the other hand, *B. viridis* can be grown only under light anoxic conditions, and is not genetically amenable. Study of the BChl *b*-containing RC complex of *B. viridis* has not progressed very far since the crystal structure of the RC was solved at the atomic level by Deisenhofer et al.^8^, for which they were awarded the Nobel Prize in 1988. The biosynthetic step to form the characteristic C8-ethylidene group on BChl *b* had remained unknown until our *in vitro* enzymatic assays revealed the enzyme responsible for the ethylidene formation^9^. Canniffe and Hunter later investigated the enzymatic activity by *in vivo* complementation experiments^10^.

Biosynthetic pathways for BChl *a* and BChl *b* are branched at the step catalyzed by chlorophyllide *a* oxidoreductase (COR)^9^. COR, a nitrogenase-like enzyme, is composed of three subunits: BchX is an electron-donating component, and BchY and BchZ form a heterotetramer (BchYZ) and work as a catalytic component. We have demonstrated that COR of the BChl *a*-producing bacterium *R. capsulatus* (*a*-COR) has dual functions: the 8-vinyl reduction of 8-vinyl-chlorophyllide (8V-Chlide) *a* and the C7 = C8 double bond reduction of resultant chlorophyllide (Chlide) *a*, forming 3-vinyl-bacteriochlorophyllide (3V-BChlide) *a* as a product (Fig. 1)^9^,^11^,^12^. We have also revealed that, in contrast, COR from BChl *b*-producing *B. viridis* (*b*-COR) recognizes only 8V-Chlide *a*, not Chlide *a*, as its substrate and catalyzes the direct formation of 3V-BChlide *b* ( = BChlide *g*) possessing the C-8 ethylidene group (Fig. 1)^9^. It is noteworthy that the two types of CORs are well conserved in their amino acid sequences, up to 89% similarities^9^. The plasticity of the nitrogenase-like enzyme not only causes the committed pathways for biosynthesis of BChls *a* and *b*, but also show a unique example of subtle amino acid substitutions in enzyme(s) that results in profound changes between the energetics of photosystems with BChl *a* and BChl *b*.

Reduction of the 8-vinyl group of chlorophyll intermediates is performed by 8-vinyl reductase, also called divinyl reductase (DVR)^6^,^13^,^14^,^15^. DVR is divided into two types: one is plant-type BciA using NADPH as electron donors, and the other is cyanobacterial-type BciB using ferredoxin as electron donors^13^. In addition, as mentioned above, COR of *R. capsulatus* can work like DVR, i.e., it has the 8-vinyl-reduction ability^9^,^12^. Until the function of BciA was revealed in 2007^14^, BchJ had been considered to be DVR^16^. Indeed, a *bchJ*-deletion mutant of *R. capsulatus* accumulated a large amount of an intermediate pigment, 8-vinyl-protochlorophyllide (8V-PChlide) *a*^16^. BchJ is known to be involved in BChl biosynthesis, although how is still unclear.

In this study, we introduced and overexpressed BchYZ of *B. viridis* in a series of *R. sphaeroides* mutant strains. The mutant of *R. sphaeroides* lacking functions of intrinsic BciA and COR and overexpressing extrinsic BchYZ of *B. viridis* produced BChl *b* under dark microoxic conditions. We also constructed the *R. sphaeroides* mutant lacking BciA and BchJ and overexpressing BchYZ of *B. viridis*, resulting in the strain producing both BChl *a* and BChl *b*. The proposed function of BchJ will be discussed.

## Results and discussion

### Construction and pigment analysis of the platform *R. sphaeroides* mutant strains

The wild-type strain of *R. sphaeroides* was used as a host strain to construct the single mutants, Δ*bchZ* and Δ*bchJ* (Fig. S1). The Δ*bciA/bchZ* and Δ*bciA/bchJ* mutants of *R. sphaeroides* were constructed in the same manner using the Δ*bciA* mutant^12^ as a host strain. Analytical PCR experiments confirmed that the *bchZ* or *bchJ* allele was completely segregated in each mutant strain (Figs. S1CD, details of the analytical PCR are described in Supplementary Information).

**Figure 1:**
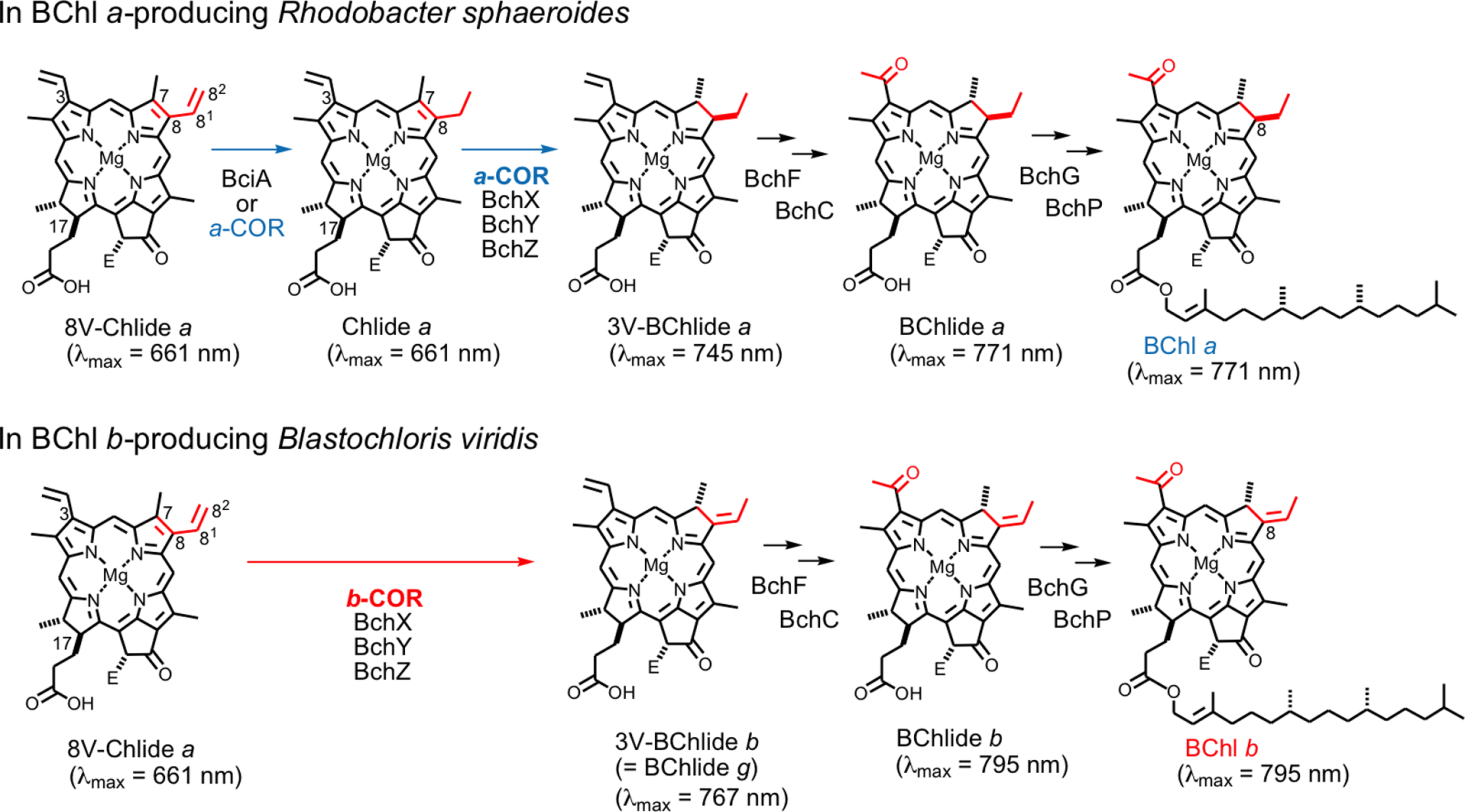
Late steps of BChl *a* biosynthesis in *R. sphaeroides* (top) and of BChl *b* biosynthesis in *B. viridis* (bottom). The presented λ_max_ peak positions are values obtained in diethyl ether. E = COOCH_3_.

**Figure 2:**
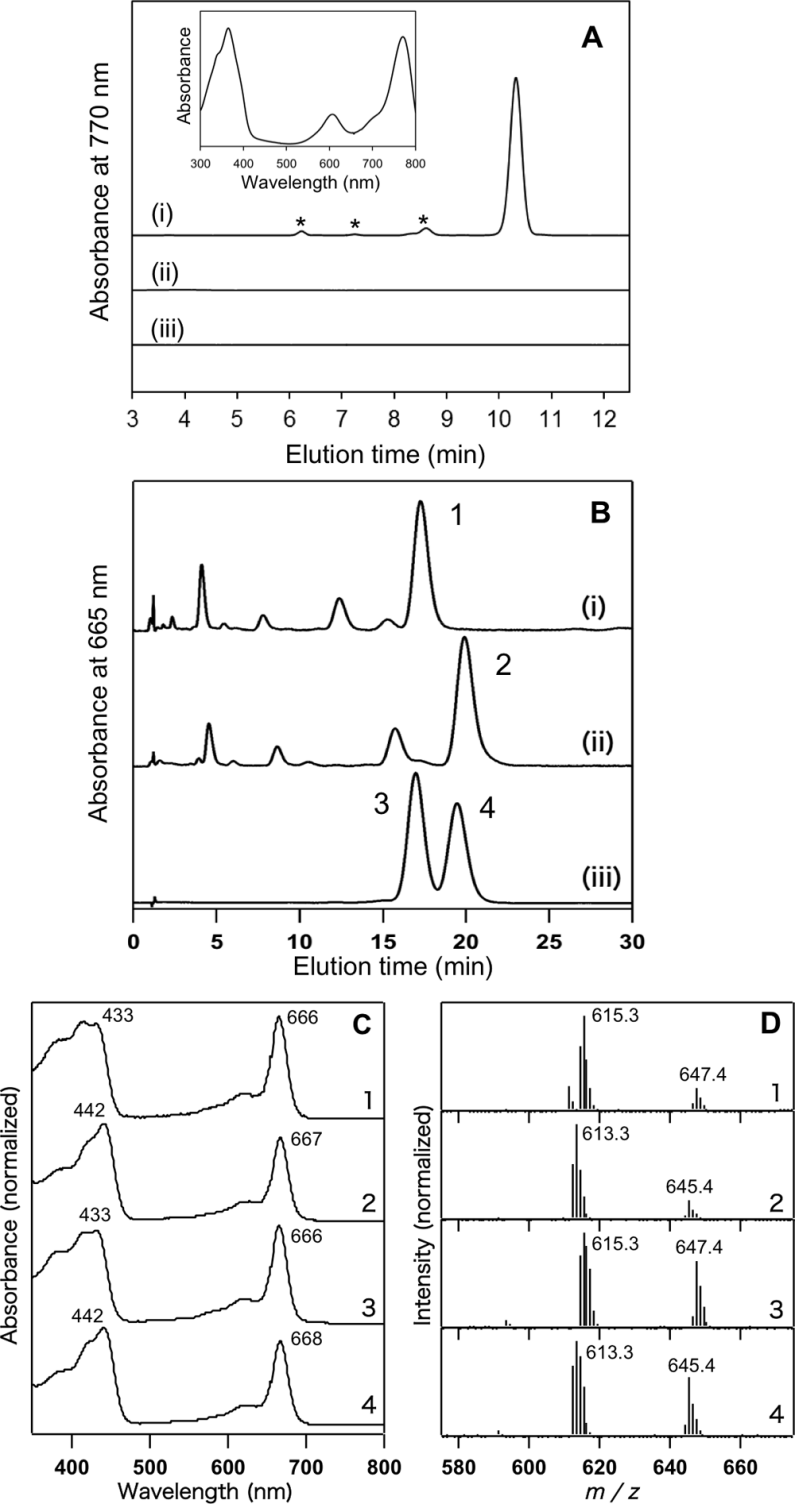
Reverse-phase HPLC-MS analysis of pigments extracted from the wild-type, Δ*bchZ*, and Δ*bciA*/*bchZ* strains. (A, traces *i*-*iii*) HPLC elution profiles of hydrophobic pigments from the wild-type, Δ*bchZ,* and Δ*bciA*/*bchZ* strains, respectively, monitored at 770 nm. (A, *inset*) In-line absorption spectrum of the elution peak at 10.5 min shown in trace *i*. Minor elution peaks shown with asterisks in Fig. 2A are BChl *a* esterified with unreduced (geranylgeranyl, dihydrogeranylgeranyl, and tetrahydrogeranylgeranyl) tails at the C17 position, according to the previous study^17^. (B, traces *i* and *ii*) HPLC elution profiles of hydrophilic pigments extracted from the Δ*bchZ* and Δ*bciA*/*bchZ* mutants, respectively, monitored at 435 nm. (B, trace *iii*) HPLC elution profile of pigment standard mixtures containing Chlide *a* (peak 3) and 8V-Chlide *a* (peak 4), monitored at 435 nm. (C) In-line absorption spectra of peaks 1-4 shown in Fig. 2B. (D) In-line mass spectra of peaks 1–4 shown in Fig. 2B.

Figure 2A shows high performance liquid chromatography (HPLC) elution profile of the authentic BChl *a* extracted from the *R. sphaeroides* wild-type strain (trace *i*). *R. sphaeroides* is known to mainly produce BChl *a* esterified with phytol as a hydrocarbon tail (trace *i*, peak at 10.5 min), although it contains trace amounts of BChl *a* esterified with unreduced (geranylgeranyl, dihydrogeranylgeranyl, and tetrahydrogeranylgeranyl) tails (trace *i*, asterisks)^17^. In-line absorption spectrum of the elution peak at 10.5 min represents a typical BChl *a* absorption spectrum with λ_max_ at 770 nm (Fig. 2A, *inset*). Pigments of *R. sphaeroides* mutant strains grown under dark microoxic conditions were also extracted and analyzed by HPLC. The Δ*bchZ* and Δ*bciA/bchZ* mutants did not produce any hydrophobic BChl compound (Fig. 2A, traces *ii* and *iii*), but accumulated hydrophilic Chlide-like pigments (Fig. 2B, traces *i* and *ii*). The Chlide-like component in the Δ*bchZ* mutant (Fig. 2B, peak 1) eluted at the same time as the standard Chlide *a* did (peak 3). In-line absorption and mass spectra of the pigment (Figs. 2CD, traces 1) were identical to those of the standard Chlide *a* (Figs. 2CD, trace 3). This indicates that the Δ*bchZ* mutant lacks the function of COR and therefore accumulates Chlide *a*, an intermediate pigment in biosynthetic pathways for BChl *a* (see Fig. 1). The Chlide-like hydrophilic component from the Δ*bciA*/*bchZ* mutant (Fig. 2B, peak 2) eluted 3-min later than Chlide *a* (peak 3) but at the same time as the standard 8V-Chlide *a* (peak 4). The in-line absorption spectrum of peak 2 (Fig. 2C, trace 2) was almost identical to that of the standard 8V-Chlide *a* (Fig. 2C, trace 4). Also, the in-line mass spectrum of peak 2 (Fig. 2D, trace 2) was almost identical to that of the standard 8V-Chlide *a* (Fig. 2D, trace 4). These indicate that the Δ*bciA*/*bchZ* mutant accumulates 8V-Chlide *a*, a precursor for Chlide *a* (Fig. 1).

### *In vitro* COR activity assays using heterologous BchX and BchYZ

**Figure 3:**
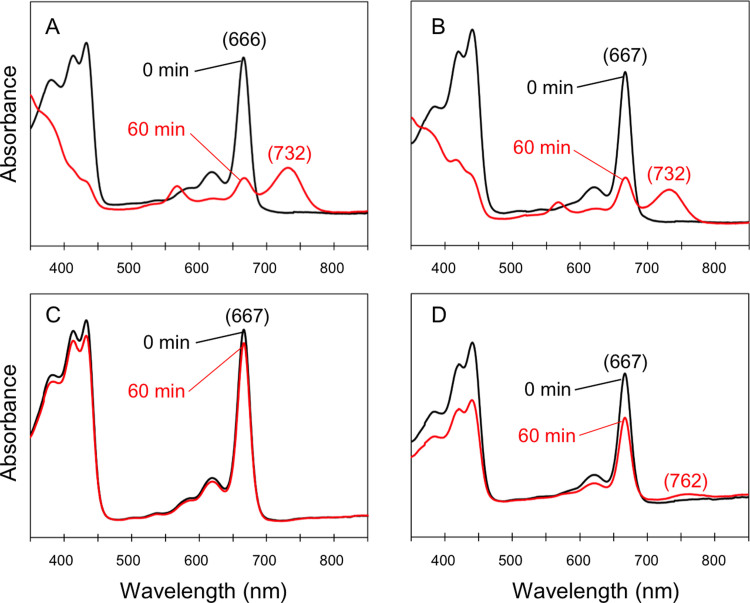
Changes in electronic absorption spectra of COR assay mixtures. Absorption spectra were recorded in 80% acetone (20% aqueous buffer). The *b*-X and *a*-YZ components were mixed with Chlide *a* (A) and 8V-Chlide *a* (B). The *a*-X and *b*-YZ components were mixed with Chlide *a* (C) and 8V-Chlide *a* (D). The Q_y_ absorption peaks of the substrates and products are shown in parentheses.

COR is composed of three subunits, BchX, BchY, and BchZ. BchX is an electron-donating component, and BchYZ works as a catalytic component^11^. In the previous study, we constructed plasmids to overexpress BchX and BchYZ components in *E. coli*^9^. In this study, we separately purified BchX and BchYZ of *R. capsulatus* (*a*-X and *a*-YZ) and those of *B. viridis* (*b*-X and *b*-YZ), and assayed COR activities *in vitro* in the heterologous combination. COR activities were assayed by absorption changes in 80% acetone extracts, according to our previous studies^9^,^18^. The heterologous combination of *b*-X and *a*-YZ was mixed with Chlide *a* (Fig. 3A). After 60-min incubation, the substrate peak from Chlide *a* at 666 nm decreased, concomitantly with the appearance of a new peak from the assay product of 3V-BChlide *a* at 732 nm (Fig. 3A). When the *b*-X and *a*-YZ components were mixed with 8V-Chlide *a*, the same phenomenon was observed (Fig. 3B). These results are almost identical to the previous results of assays using all BchXYZ components from a BChl *a*-producing bacterium^9^. As another combination, *a*-X and *b*-YZ components were mixed with Chlide *a*, then no product peak was observed in the region of 700–750 nm (Fig. 3C). But the assay mixture of *a*-X and *b*-YZ with 8V-Chlide *a* showed a new peak of the assay product of 3V-BChlide *b* ( = BChlide *g*) at 762 nm (Fig. 3D). These also support the results in the previous study using all BchXYZ components from *B. viridis* for the assay^9^. These assay results clearly indicate that BchX is able to transfer electrons to heterologous BchYZ components to form active CORs, and that the pattern of catalytic activities of *a*- and *b*-type CORs is BchYZ-dependent.

### *R. sphaeroides* recombinant strains overexpressing BchYZ of *B. viridi*s

Taking the results of the *in vitro* enzymatic assays using heterologous COR components into consideration, we introduced only the *bchYZ* genes of *B. viridis* into the wild-type and mutant strains of *R. sphaeroides*. The plasmid pJ7-BvYZ-Gm carrying the *bchYZ* genes of *B. viridis* was incorporated into the wild-type, Δ*bchZ*, and Δ*bciA*/*bchZ* strains of *R. sphaeroides*, resulting in strains named WT+BvYZ, Δ*bchZ*+BvYZ, and Δ*bciA/bchZ*+BvYZ, respectively. After conjugation, transconjugant colonies on gentamycin^r^ selective plates were re-streaked on selective plates two times, and then a single colony was picked up and grown in liquid medium. The cultures grown under dark microoxic conditions were harvested, and pigments were extracted and analyzed by HPLC.

**Figure 4:**
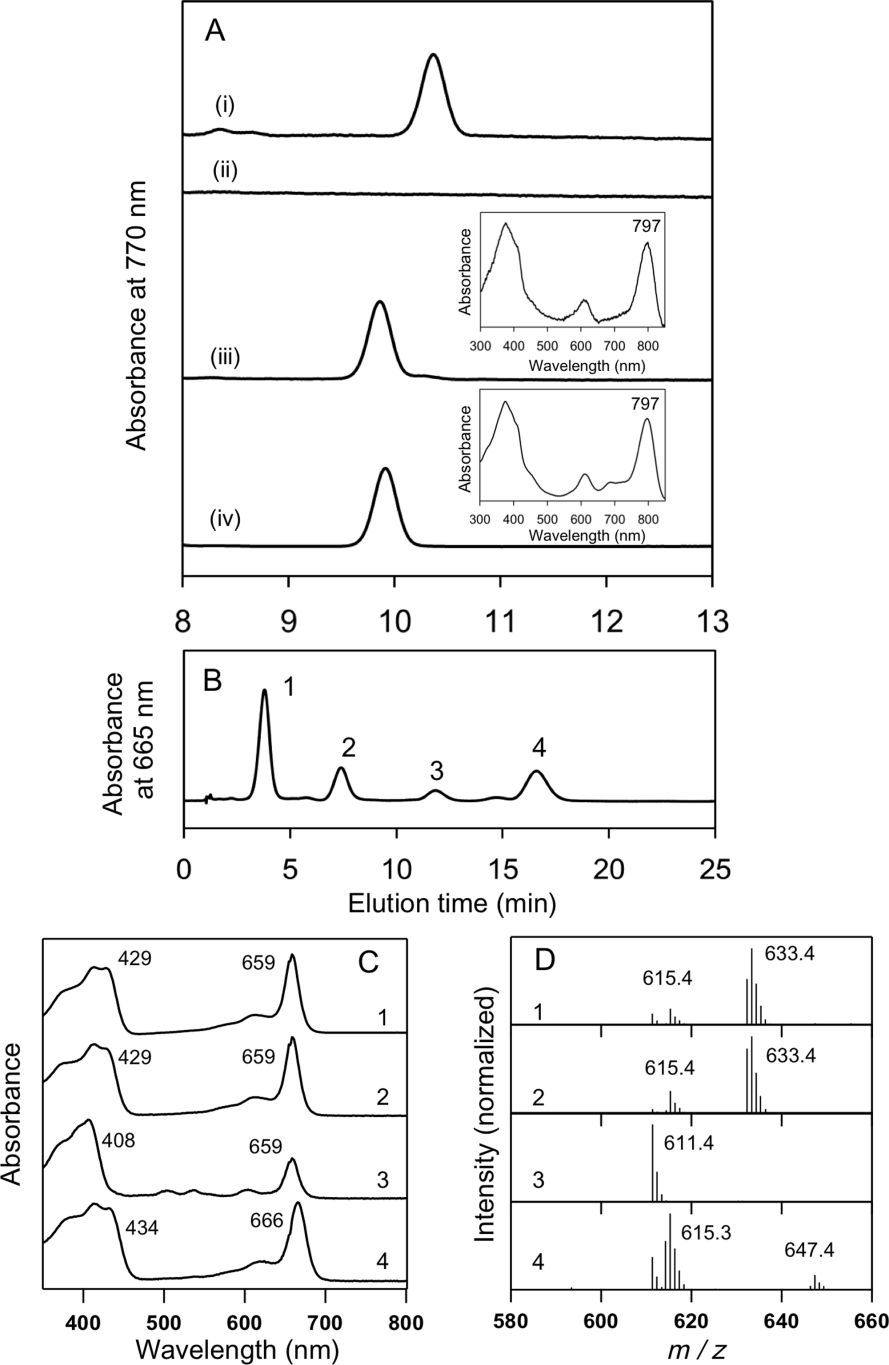
Reverse-phase HPLC-MS analysis of pigments extracted from the *R. sphaeroides* mutant strains expressing *B. viridis* BchYZ and from the wild type of *B. viridis*. (A, traces *i*-*iv*) HPLC elution profiles of hydrophobic pigments from WT+BvYZ, Δ*bchZ*+BvYZ, Δ*bciA*/*bchZ*+BvYZ, and the wild-type *B. viridis*, respectively, monitored at 770 nm. (A, *top and bottom insets*) Electronic absorption spectra of the pigments from the Δ*bciA*/*bchZ*+BvYZ mutant and *B. viridis*, respectively, in methanol. The pigment elution peaks shown in traces *iii* and *iv* of Fig. 4A were collected by preparative HPLC, and the absorption spectra were measured with a Hitachi U-3500 spectrophotometer (Tokyo, Japan). (B) A HPLC elution profile of hydrophilic pigments accumulated in the Δ*bchZ*+BvYZ cultures monitored at 665 nm. (C) In-line absorption spectra of peaks 1-4 shown in Fig. 4B. (D) In-line mass spectra of peaks 1-4 shown in Fig. 4B.

The pigment extracted from the WT+BvYZ strain showed the same elution time (Fig. 4A, trace *i*) as the authentic BChl *a* extracted from wild type did (Fig. 2A, trace *i*), indicating that the intrinsic *a*-COR still dominantly works in the mutant strain even when *B. viridis* BchYZ proteins are overexpressed. The Δ*bchZ*+BvYZ strain did not produce any hydrophobic BChl compound (Fig. 4A, trace *ii*), again even though *B. viridis* BchYZ components were overexpressed. This suggests that the intrinsic BciA of *R. sphaeroides* dominantly reacts with 8V-Chlide *a*, and therefore 8V-Chlide *a*, a suitable substrate for the *B. viridis* BchYZ, is not available any longer. The suggestion can be proved by making the Δ*bciA/bchZ*+BvYZ mutant. Figure 4A, trace *iii*, shows that the BChl component extracted from the Δ*bciA/bchZ*+BvYZ strain had an elution time at about 10 min, 0.5-min earlier than that of BChl *a.* The authentic BChl *b* extracted from *B. viridis* showed the same retention time (Fig. 4A, trace *iv*). The BChl components eluting at 10 min from the Δ*bciA/bchZ*+BvYZ mutant and *B. viridis* were collected by preparative HPLC and electronic absorption spectra were measured. The absorption spectra of the collected pigments were identical and both showed λ_max_ at 797 nm (Figs. 4A, *insets*), clearly indicating that the Δ*bciA/bchZ*+BvYZ strain produced BChl *b*. These results indicate that the loss of DVR and the replacement of intrinsic BchYZ catalytic components by BchYZ of BChl *b*-producing bacteria are required in order to make *R. sphaeroides* produce BChl *b*: i.e., exchanging all of the BchXYZ subunits is not necessary. The average amount of BChl *b* molecules produced in the Δ*bciA/bchZ*+BvYZ mutant was ~30 mg per 1-L culture. The *R. sphaeroides* mutant can grow and synthesize BChl *b* even under dark microoxic conditions, and it could be a good platform for industrial production of BChl *b*.

Although the Δ*bchZ*+BvYZ strain produced no hydrophobic BChl compound, it accumulated hydrophilic Chlide-like pigments (Fig. 4B). The HPLC elution profile of hydrophilic Chlide-like pigments extracted from the Δ*bchZ*+BvYZ mutant showed one major elution peak and three minor peaks (Fig. 4B). In-line absorption spectra of peaks 1 and 2 were identical, and both showed the Q_y_ absorption band at 659 nm and the Soret band at 429 nm (Fig. 4C, traces 1 and 2), 7-nm and 4-nm blue-shifted from those of the standard Chlide *a*, respectively (Fig. 2C, trace 3). Also, the pigments eluted as peaks 1 and 2 had a mass of 633.4 (Fig. 4D, trace 1 and 2), 18 mass units larger than the mass of Chlide *a* (615.3, Fig. 2D, trace 3). These results, together with the presence of a fragment 615.4 mass peak (Fig. 4D, traces 1 and 2), and with the fact that peaks 1 and 2 eluted earlier than the standard Chlide *a* (Fig. 2B, peak 3), indicate that these two elution peaks are ascribable to 3-(1-hydroxyethyl)-Chlide *a* with 3^1^*R*- and 3^1^*S*-configurations. In-line absorption spectrum of the minor peak 3 in Fig. 4B showed the Soret band at 408 nm (Fig. 4C, trace 3), about 20-nm blue-shifted from those of peaks 1 and 2 (traces 1 and 2). The clear appearance of Q_x_ bands at 500–550 nm were observed in the absorption spectrum of peak 3 (Fig. 4C, trace 3). The pigment eluting as peak 3 gave *m*/*z* = 611.4 as its parent mass peak (Fig. 4D, trace 3), which is 22 mass units smaller than that of 3-(1-hydroxyethyl)-Chlide *a* (Fig. 4D, traces 1 and 2). These suggest that the pigment eluting as peak 3 is 3-(1-hydroxyethyl)-pheophorbide *a* lacking the central magnesium*.* In-line absorption and mass spectra as well as elution time of peak 4 (Figs. 4CD, traces 4, and Fig. 4B) were almost identical to those of the standard Chlide *a* (Figs. 2CD, traces 3, and Fig. 2B, peak 3), indicating that the pigment eluting as peak 4 was Chlide *a*.

These results indicate that the Δ*bchZ*+BvYZ mutant mainly accumulated 3-(1-hydroxyethyl)-Chlide *a.* On the other hand, Canniffe and Hunter^10^ recently constructed a similar mutant using a different method, homologous gene recombination, and reported different results. The mutant of *R. sphaeroides* they made, in which all the intrinsic *bchXYZ* genes were deleted and then the exogenous *bchXYZ* genes of *B. viridis* were incorporated into the genome, produced BChl *a*. However, our previous study^9^ has clearly indicated that the suitable substrate for the *B. viridis* COR proteins is 8V-Chlide *a*, not Chlide *a*, and the present study also supports the idea that BchYZ components of *B. viridis* do not react with Chlide *a* (Figs. 3 and 4). Amino acid sequences of *a*-COR and *b*-COR proteins have very high similarities^9^. There is a possibility that their mutant could have second mutations in the amino acid sequence that changes the hydrogenation mode of CORs, from 1,4-addition to 1,2-addition. This could happen by the change of even a few amino acid residues. Incorporated exogenous *bchXYZ* genes in the genome of their mutant were only confirmed by PCR amplification, but the sequence of the PCR product was not sequenced^10^. It would be very interesting if the incorporated *bchXYZ* were sequenced and amino acid substitutions were found.

The hydration of the C3 vinyl group to complete BChl biosynthesis is catalyzed by BchF hydratase (Fig. 1)^19^. In the Δ*bchZ* mutant, the pigment biosynthetic pathway was almost suspended at the Chlide-*a*/BchXYZ step, although the mutant probably produced a small amount of 3-(1-hydroxyethyl)-Chlide *a*; in-line absorption and mass spectra of a minor elution peak from the Δ*bchZ* mutant (Supplementary Fig. S2) are almost identical to those of 3-(1-hydroxyethyl)-Chlide *a* from the Δ*bchZ*+BvYZ strain (Fig. 4CD, traces 1). On the other hand, the Δ*bchZ*+BvYZ mutant mainly accumulated 3-(1-hydroxyethyl)-Chlide *a* (Fig. 4B, peak 1): it seemed that BchF carried out its catalytic reaction in the mutant rather than in the Δ*bchZ* mutant. The difference between the two mutants is only the presence of overexpressed BchYZ of *B. viridis* in the Δ*bchZ*+BvYZ mutant, and this therefore would be the reason for the difference in accumulated pigment intermediates. The overexpressed *B. viridis* BchYZ proteins would probably sequester Chlide *a* in the Δ*bchZ*+BvYZ mutant, but no reaction happens because the suitable substrate for *b*-COR to achieve its reaction is 8V-Chlide *a*^9^. There might then be a substrate exchange from BchYZ to BchF, which forms a 1-hydroxyethyl group at the C3 position.

### Electronic absorption spectrum of the BChl *b*-producing *R. sphaeroides* strain

**Figure 5:**
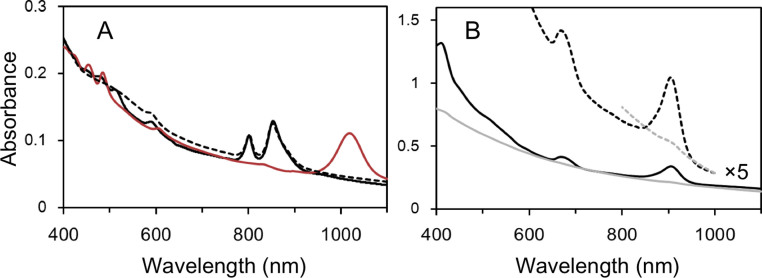
Electronic absorption spectra of *R. sphaeroides*, *B. viridis*, and the Δ*bciA*/*bchZ*+BvYZ mutant. (A) Electronic absorption spectra of *R. sphaeroides* cells grown under light anoxic (*black solid line*) and dark microoxic conditions (*black dashed line*) and *B. viridis* cells grown under light anoxic conditions (*red line*). (B) Electronic absorption spectrum of membrane suspensions (*black lines*) and whole cells (*gray lines*) of the Δ*bciA*/*bchZ*+BvYZ strain grown under dark microoxic conditions. Cells and membrane suspensions were suspended in 10 mM Tris-HCl buffer, pH 7.5.

Figure 5A shows electronic absorption spectra of *R. sphaeroides* and *B. viridis* cells. *R. sphaeroides* has RC-LH1 (~875 nm) and LH2 (~800 and ~850 nm) antenna complexes^4^. On the other hand, *B. viridis* does not have LH2 but has only RC-LH1 complex (~1015 nm)^5^. Membrane suspensions from aerobically-grown Δ*bciA/bchZ*+BvYZ cultures showed an absorption band at about 900 nm, but did not show any absorption peak over the 900-nm wavelength region (Fig. 5B). This implies that the large blue-shifted absorption band of the *B. viridis* RC-LH1 complex is mainly caused by polypeptide environments surrounding pigment cofactors, as well as by change of the embedded pigment (from BChl *a* to BChl *b*). Note that the Δ*bciA/bchZ*+BvYZ mutant was not able to grow under light anoxic conditions.

### Function of BchJ as an enhancer for the COR activity

**Figure 6:**
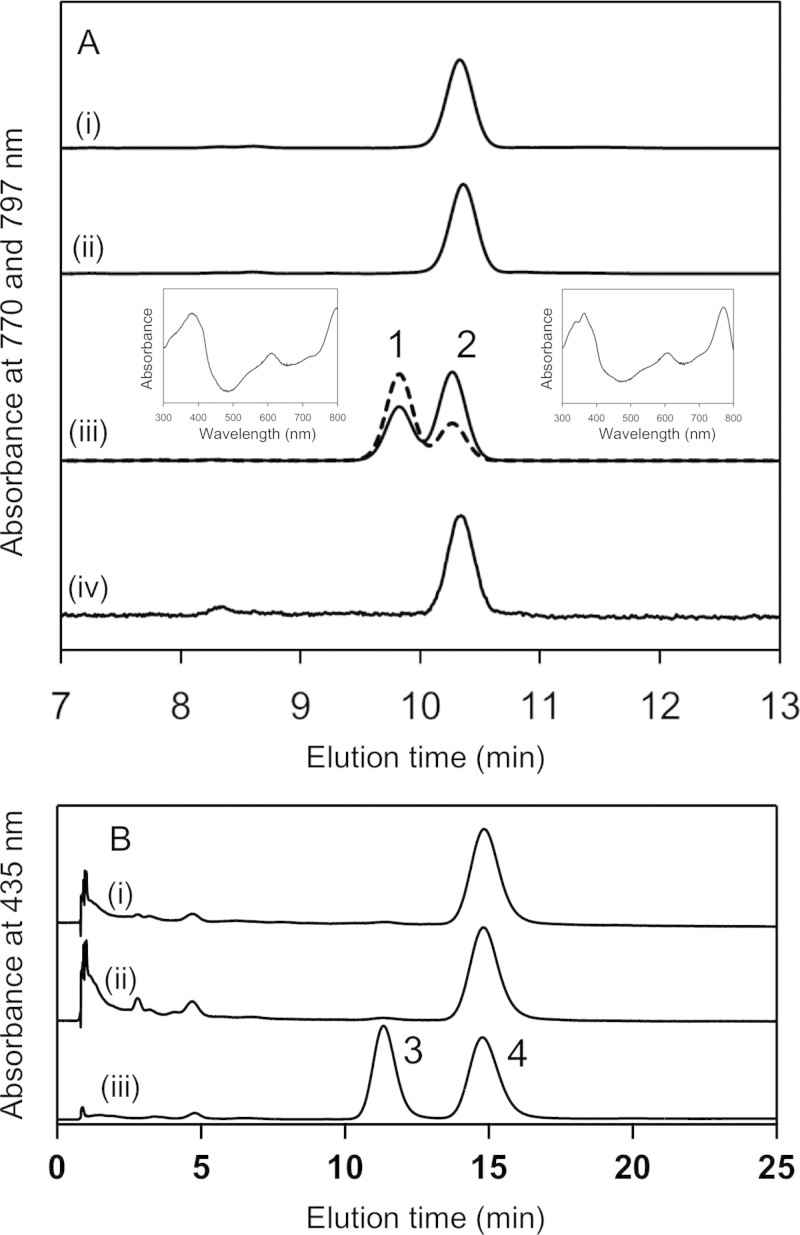
Reverse-phase HPLC analysis of pigments extracted from the Δ*bchJ*-relevant strains of *R. sphaeroides*. (A, traces *i*-*iv*) HPLC elution profiles of hydrophobic pigments extracted from the Δ*bchJ,* Δ*bchJ/bciA,* Δ*bchJ/bciA*+BvYZ, Δ*bciA*+BvYZ mutant strains, respectively, monitored at 770 nm (*solid line*) and at 797 nm (*dashed line*). In-line absorption spectra of peak 1 (*left inset*) and peak 2 (*right inset*) were measured with a Shimadzu photodiode-array spectrophotometer detector (SPD-M20A) equipped in a Shimadzu HPLC system. Note that the detection limit of the spectrophotometer detector for the long wavelength is 800 nm. (B, traces *i*-*iii*) HPLC elution profiles of hydrophilic pigments extracted from the Δ*bchJ* and Δ*bchJ/bciA* mutant strains and of a mixture of standards PChlide *a* (peak 3) and 8V-PChlide *a* (peak 4), respectively, monitored at 435 nm.

BchJ has long been considered to be involved in BChl *a* biosynthesis, but its function is still uncertain. Previous study demonstrated that Δ*bchJ* mutant of the purple bacterium *R. capsulatus* accumulated 8V-PChlide *a*^16^, and therefore BchJ was first thought to work as DVR. Later, Chew and Bryant showed that Δ*bchJ* mutant of the green sulfur bacterium *Chlorobaculum tepidum* also accumulated 8V-PChlide *a* in the spent medium, but their detailed analysis found that the mutant still produced a small amount of normal (8-ethylated) BChl *a*, and concluded that BchJ is not DVR^14^. Thus, the function of BchJ in BChl biosynthesis has been enigmatic. Here, we investigated the function of BchJ by using the overexpression system of exogenous COR in *R. sphaeroides*. We first made the deletion mutant of the *bchJ* gene of *R. sphaeroides*. HPLC elution profiles of pigments extracted from the Δ*bchJ* mutant demonstrated that this mutant produced a small amount of a hydrophobic BChl pigment (Fig. 6A, trace *i*) and a hydrophilic pigment (Fig. 6B, trace *i*). Compared to elution profiles of the standards of BChl *a* (Fig. 2A, trace *i*) and 8V-PChlide *a* (Fig. 6B, peak 4), the two pigments from the Δ*bchJ* mutant of *R. sphaeroides* are ascribable to BChl *a* and 8V-PChlide *a*, respectively, as shown in the Δ*bchJ* mutants previously reported^14^,^15^. We next constructed the Δ*bchJ*/*bciA* double mutant, which showed the same phenotype on pigment compositions (Figs. 6AB, traces *ii*) as that of the single Δ*bchJ* mutant (Figs. 6AB, traces *i*). This suggests that the intrinsic *a*-COR present in the double mutant works as DVR, as previously reported^9^,^12^, instead of the deleted BciA.

The plasmid pJ7-BvYZ-Gm carrying the *bchYZ* genes of *B. viridis* was transformed into the Δ*bchJ*/*bciA* and Δ*bciA* mutant strains of *R. sphaeroides*, resulting in strains termed Δ*bchJ*/*bciA*+BvYZ and Δ*bciA*+BvYZ, respectively. HPLC elution profile of the Δ*bchJ*/*bciA*+BvYZ mutant exhibited two elution peaks of hydrophobic BChl pigments (Fig. 6A, trace *iii*). The two pigments (peaks 1 and 2 in Fig. 6A) had the same elution times as those of the BChl *b* (Fig. 4A, trace *iv*) and BChl *a* standards (Fig. 2A, trace *i*), respectively. In-line absorption spectra of peaks 1 and 2 showed λ_max_ at 797 nm and 770 nm, respectively (Fig. 6A, *insets*). These results indicate that the Δ*bchJ*/*bciA*+BvYZ mutant produces both BChls *a* and *b*. This is the first example of a mutant of anoxygenic photosynthetic bacteria having both BChls *a* and *b*.

The Δ*bciA*+BvYZ mutant lacking BciA accumulated only BChl *a* and did not show the elution peak of BChl *b* (Fig. 6A, trace *iv*), although 8V-Chlide *a* could be potentially available as the substrate for the overexpressed *B. viridis* BchYZ in the mutant, indicating that the activity of the intrinsic BchYZ is much faster than that of the overexpressed *B. viridis* BchYZ in the mutant. On the other hand, the activities of the intrinsic and extrinsic BchYZ seemed to be almost equal in the Δ*bchJ*/*bciA*+BvYZ mutant lacking BchJ. These results from the two mutants suggest that BchJ in *R. sphaeroides* facilitates the catalytic activity of only the intrinsic *a*-COR. Further investigation is still needed to learn whether the exact function of BchJ is as a substrate carrier, a scaffold protein to form tertiary complexes, or a chaperon for pigment biosynthesis proteins. Because the amounts of BChls *a* and *b* produced in the Δ*bchJ*/*bciA*+BvYZ mutant were almost the same (Fig. 6A, trace *iii*), both intrinsic *a*-COR and exogenous *b*-COR catalytic components were likely to be almost equally functional. This implies that BchJ may form a tertiary complex with pigment substrates and pigment biosynthesis enzymes. Therefore, in the Δ*bchJ* background, the *B. viridis* BchYZ proteins could access pigment substrates to a degree equal to *a*-COR (*R. sphaeroides* BchYZ).

Sawicki and Willows^20^ suggested that BchJ might play a role as a porphyrin carrier working at the steps between BchIDH (magnesium chelatase) and BchM (Mg-protoporphyrin IX monomethyl esterase) in the early stages of BChl *a* biosynthesis of *R. capsulatus*. In addition, preliminary experiments have shown that BchJ enhances the catalytic activity of dark-operative PChlide *a* oxidoreductase working at the BChl *a* biosynthetic step followed by COR (Yamanashi, K. and Fujita, Y., personal communication). Taking these into account, it is highly likely that BchJ are involved in most of the BChl *a* biosynthetic pathways to facilitate enzymes working in the pigment biosynthesis.

## Methods

### Construction of the Δ*bchZ* and Δ*bciA/bchZ* mutants of *R. sphaeroides*

The wild-type strain J001 and the Δ*bciA* mutant of *R. sphaeroides*, constructed in a previous study^12^, were used as host strains to construct the Δ*bchZ* and Δ*bciA*/*bchZ* mutants, respectively. The plasmid pJSC-bchZ-Sm used for the insertional inactivation of *bchZ* was constructed as follows.

The *aadA* gene, conferring resistance to streptomycin and spectinomycin, was amplified from plasmid pHP45Ω^21^ by PCR using a primer set, aadA-F (Fig. S1A, shown as primer *i*) and aadA-R (primer *ii*). Primer positions and sequences are presented in Fig. S1 and Table S1, respectively. The *bchZ* gene and a portion of *bchY* were amplified from the genomic DNA of *R. sphaeroides* by PCR using bchZ-F (primer *iii*) and bchZ-R (primer *iv*) primers. The PCR reactions were performed with KOD-plus DNA polymerase (TOYOBO, Osaka, Japan). The DNA fragment containing *bchZ* was sub-cloned into pTA2 by the TA cloning method (TOYOBO), yielding pTA-bchZ (Fig. S1A). To amplify a DNA fragment from pTA-bchZ without the large inner portion of *bchZ*, the plasmid was used as the template for the inverse PCR with primers bchZ-inf-FI (primer *v*) and bchZ-inf-RI (primer *vi*). The DNA fragment and the above-mentioned PCR product of the *aadA* gene were ligated with an In-Fusion HD cloning kit (Clontech, USA), yielding pTA-bchZ-Sm (Fig. S1A). The DNA fragment containing the partial *bchZ* gene disrupted by the *aadA* gene was amplified from the plasmid pTA-bchZ-Sm by PCR using primers bchZ-inf-FII (primer *vii*) and bchZ-inf-RII (primer *viii*), and sub-cloned into the SmaI restriction sites of the pJSC vector^22^ by the In-Fusion technique, producing pJSC-bchZ-Sm. The plasmid pJSC is a chloramphenicol-resistant suicide vector and has the *sacB* gene encoding the levansucrase; the expression of *sacB* in the presence of sucrose is lethal for most of the Gram-negative bacteria^23^.

The plasmid pJSC-bchZ-Sm was transformed into the mobilizing *E. coli* strain S17-1 λ-*pir*^24^. By conjugation method with the *E. coli* S17-1 strain^22^, pJSC-bchZ-Sm was transferred into the wild-type strain and Δ*bciA* mutant of *R. sphaeroides*. Colonies grown in the presence of 5% sucrose, 50 μg/mL streptomycin, and 100 μg/mL rifampicin were selected as double-crossover candidates, and the chromosomal insertion into *bchZ* by the *aadA* gene was confirmed by analytical PCR using bchZ-comf-F (Figs. S1A, primer *ix*) and bchZ-comf-R (primer *x*) primers (see Supplementary text for details of the analytical PCR experiments). The obtained Δ*bchZ* and Δ*bciA/bchZ* mutants were grown under dark microoxic conditions in the PYS medium^25^ at 30°C. These strains and the wild-type strain of *R. sphaeroides* were used as hosts to incorporate the *bchYZ* genes of *B. viridis*.

### Construction of the Δ*bchJ* and Δ*bciA/bchJ* mutants of *R. sphaeroides*

The wild-type and Δ*bciA* mutant strain of *R. sphaeroides* were used as host strains to construct the Δ*bchJ* and Δ*bciA*/*bchJ* mutants, respectively. The plasmid pJSC-bchJ-Sm for the insertional inactivation of the *bchJ* gene in *R. sphaeroides* was constructed as follows.

A 1.61-kbp DNA fragment containing *bchJ* was amplified from the genome of *R. sphaeroides* using primers, bchJ-F (Fig. S1B, primer *xi*) and bchJ-R (primer x*ii*). The PCR product containing the *bchJ* gene was sub-cloned into the plasmid pTA2, yielding pTA-bchJ (Fig. S1B). To amplify a DNA fragment from pTA-bchJ without the inner portion of *bchJ*, the plasmid was used as the template for the inverse PCR with primers, bchJ-inf-FI (primer *xiii*) and bchJ-inf-RI (primer *xiv*). The resulting PCR product and the *aadA* gene amplified from plasmid pHP45Ω mentioned above were ligated using the In-Fusion HD Cloning Kit, creating pTA-bchJ-Sm (Fig. S1B). The plasmid pTA-bchJ-Sm was used as the template for PCR using primers, bchJ-inf-FII (primer *xv*) and bchJ-inf-RII (primer *xvi*). The amplified DNA fragment was cloned into the SmaI site of pJSC by the In-Fusion cloning technique, yielding the pJSC-bchJ-Sm plasmid. The plasmid pJSC-bchJ-Sm was transformed into *E. coli* S17-1 λ-*pir*, and then into the wild-type strain and Δ*bciA* mutant of *R. sphaeroides* by the conjugation method in order to create the Δ*bchJ* and Δ*bciA/bchJ* mutants, respectively. Streptomycin-resistant colonies grown in the presence of 5% sucrose were selected as double-crossover candidates, and the chromosomal insertion into *bchJ* by the *aadA* gene was confirmed by analytical PCR using bchJ-comf-F (primer *xvii*) and bchJ-comf-R (primer *xviii*) primers (see Supplementary text and Fig. S1D).

### Construction of the *R. sphaeroides* strains expressing BchYZ of *B. viridis*

The broad-range host vector to overexpress exogenous gene products in *R. sphaeroides* under the control of the *puc* promoter, which we designated as pJN7, was first constructed as follows. The *puc* promoter region of *R. capsulatus* was amplified by PCR using primers, ppucf6 and pjr6 (see Table S1 for primer sequences). The PCR product was digested by the restriction enzymes SalI and BamHI, and ligated into the same sites of the plasmid pBBR1MCS2^26^ carrying the kanamycin resistance, yielding pJN6. The plasmid pJN6 was further subjected to the PCR reaction using a primer set, KOBsal-f1 and KOBsal-r1. Then, the streptomycin/spectinomycin resistant cartridge was amplified from the plasmid pJN3^27^ by PCR using primers, Spc2f1 and Spc2r1. These two DNA fragments were digested by SacI and ligated together, yielding pJN7.

The *bchY* and the flanking *bchZ* genes of *B. viridis* were amplified together by PCR using primers, BvYZ-infu-F1 and BvYZ-infu-R1 (see Table S1 for primer sequences). Note that *bchY* and *bchZ* genes are usually adjacent in the genomes of phototrophic bacteria and overlapped (i.e., the start codon of *bchZ* comes before the stop codon of *bchY* in the genomes). This is the case for both *R. sphaeroides* and *B. viridis*. The amplified DNA fragment was excised from agarose gels and purified using a NucleoSpin Extract II kit (Macherey-Nagel, Duren, Germany). The purified DNA fragment containing *B. viridis* *bchY*-*bchZ* genes was sub-cloned into the BsaI restriction sites of the pJN7 plasmid with the In-Fusion HD cloning kit, yielding pJ7-BvYZ. In the plasmid, the KpnI restriction site was located in the region after the coding region of *bchZ*. The *aacC1* gene, conferring resistance to gentamycin, was amplified from the plasmid pUCGM-star^28^ by PCR using primers, Gm-JN7-F and Gm-JN7-R. The resulting PCR product containing the *aacC1* gene and the pJ7-BvYZ plasmid digested by KpnI were ligated using the In-Fusion HD cloning kit, yielding pJ7-BvYZ-Gm. The plasmid was transformed into the wild-type, Δ*bciA*, Δ*bchZ*, and Δ*bciA*/*bchZ*, Δ*bchJ*, and Δ*bciA*/*bchJ* strains of *R. sphaeroides* by triparental mating with *Escherichia coli* strain Tec5 containing the relevant plasmids^29^. Transconjugants were selected on PYS plates^25^ containing rifampicin (100 μg/ml), kanamycin (25 μg/ml), and gentamycin (10 μg/ml). Transconjugant colonies on the selective plates were grown in liquid PYS medium, and we purified plasmids from cultures and confirmed them to be pJ7-BvYZ-Gm by cutting with appropriate restriction enzymes. The resultant wild-type, Δ*bciA*, Δ*bchZ*, Δ*bciA*/*bchZ,* Δ*bchJ*, and Δ*bciA*/*bchJ* strains of *R. sphaeroides* expressing the *B. viridis* *bchYZ* genes under the control of the *puc* promoter were designated as WT+BvYZ, Δ*bciA*+BvYZ, Δ*bchZ*+BvYZ, Δ*bciA/bchZ*+BvYZ, Δ*bchJ*+BvYZ, and Δ*bciA/bchJ*+BvYZ, respectively.

### *In vitro* enzymatic assays for COR components

Plasmids to overexpress BchX and BchYZ of *R. capsulatus* and *B. viridis* in *E. coli* were constructed in the previous report^9^. Purification of these proteins, preparation of substrate pigments, and assays for COR activities were performed according to our previous studies^9^,^18^.

### HPLC conditions

The wild-type strains of *R. sphaeroides* and *B. viridis* were cultured in PYS medium^25^ at 30°C under light anoxic conditions. The mutant strains of *R. sphaeroides* were grown in PYS medium at 30°C under dark microoxic conditions. Cells were harvested by centrifugation, and pigments were extracted with acetone/methanol (1:1, vol/vol) and filtered with a PVDF 0.22-μm membrane filter. To analyze hydrophobic BChl-type pigments, reverse-phase HPLC measurements were performed using an octadecylated silica gel column (Cosmosil 5C_18_-AR-II 4.6 φ × 150 mm, 5 μm, Nacalai Tesque, Kyoto, Japan) and the mobile phase of methanol : water = 95 : 5 with the flow rate of 1.0 mL/min. The HPLC-MS system for the analysis of hydrophilic Chlide-type pigments consisted of an octadecyl–polar group–silica gel column (Inertsil ODS-EP 3.0 φ × 150 mm, 5 μm, GL Sciences Inc., Tokyo), a photodiode-array spectrophotometer detector (SPD-M20A; Shimadzu, Kyoto) and a LCMS-2010EV quadrupole mass spectrometer equipped with an electrospray ionization (ESI) probe (Shimadzu). The mobile phase was methanol : aqueous 50 mM ammonium acetate (pH 5.25) = 70 : 30 (v/v), and the flow rate was isocratic at 0.5 mL/min. The ESI-MS settings were as follows: capillary temperature; 230°C, sheath gas (N_2_) pressure; 0.1 MPa, and spray voltage; 1.5 kV (positive-ion ESI). For the HPLC-MS analysis of hydrophilic PChlide-type pigments, the following setting was applied: a polymeric octadecylated silica gel column (Inertsil ODS-P 3.0 φ × 150 mm, 5 μm, GL Sciences Inc.); eluent, methanol : acetonitrile : aqueous 50 mM ammonium acetate (pH 5.25) = 60 : 20 : 20 (v/v/v); flow rate, 0.75 mL/min.

### Electronic absorption spectra measurements of cells and membrane suspensions

Cells of *R. sphaeroides* grown under light anoxic and dark microoxic conditions and *B. viridis* grown under light anoxic conditions were harvested and suspended in 20 mM Tris-HCl (pH 7.5). Cultures of the Δ*bciA/bchZ*+BvYZ mutant grown under dark microoxic conditions were harvested by centrifugation at 9,000 × *g* for 20 min, resuspended in 20 mM Tris-HCl (pH 7.5), and disrupted by passaging three times through a French Press at 100 MPa. Unbroken cells were removed by centrifugation at 10,000 × g for 15 min, and the supernatant was used as membrane suspensions. Electronic absorption spectra were measured using a Shimadzu UV-1800 spectrophotometer (Kyoto).

## Author Contributions

Y.T. and H.T. designed the research. Y.T., J.H. and J.N. cloned genes and made inactivation and overexpression mutant strains. Y.T., H.Y. and Y.F. performed enzymatic assays. Y.T. and T.M. prepared substrates for the assay and identified assay products and pigment accumulated in the mutants by LC-MS. Y.T., Y.F. and H.T. analyzed the data and wrote the manuscript.

## Supplementary Material

Supplementary Information Supplementary Information
