# Supplementary material for: Rhodobacter sphaeroides mutants overexpressing chlorophyllide a oxidoreductase of Blastochloris viridis elucidate functions of enzymes in late bacteriochlorophyll biosynthetic pathways
Source: Sci Rep. 2015 May 15;5:9741. doi: 10.1038/srep09741 (PMC4432870; doi:10.1038/srep09741)
Supplement: Supplementary Information [file srep09741-s1.pdf]

Supplementary Information for

Rhodobacter sphaeroides mutants overexpressing chlorophyllide _a_ oxidoreductase of _Blastochloris viridis_ elucidate functions of enzymes in late bacteriochlorophyll biosynthetic pathways

Yusuke Tsukatani*, Jiro Harada, Jiro Nomata, Haruki Yamamoto, Yuichi Fujita, Tadashi Mizoguchi, and Hitoshi Tamiaki*

*****To whom correspondence should be addressed: tsukatani@elsi.jp (Y. T.) or tamiaki@fc.ritsumei.ac.jp (H. T.)

This contains Supplementary text, Figs. S1-S2, and Table S1.

Supplementary text

The mutated *bchZ* and *bchJ* loci in mutants constructed in this study were confirmed by analytical PCR experiments. PCR fragments of the *bchZ* locus when using the bchZ-comf-F and bchZ-comf-R primers are calculated to be 2.59 kbp for wild type, and to be 2.48 kbp for the Δ*bchZ* and Δ*bciA*/*bchZ* mutants. Since these fragments were actually not distinguishable in the agarose gel. (Fig. S1C, lanes 4-6), the PCR products were digested by the restriction enzyme EcoRI (lanes 7-9). The EcoRI restriction site is located at the middle of the *bchZ* gene (Fig. S1A). The digested PCR products of wild type were shown to be about 1.4 and 1.2 kbp (Fig. S1C, lane 7), but the PCR fragments from the Δ*bchZ* (line 8) and Δ*bciA*/*bchZ* (line 9) mutants were not digested by *Eco*RI. DNA sequences of the PCR fragments were determined to further confirm the mutation in the *bchZ* locus. The *bciA* loci were also confirmed using primers, sphaA-comf-F and sphaA-comf-R[1]. When using these primers, a DNA fragment from the Δ*bciA*/*bchZ* mutant (Fig. S1C, lane 3) was about 2.5 kbp, which is 1.3-kbp longer than those of the wild-type (lane 1) and Δ*bchZ* (lane 2) strains by the insertion of the kanamycin-resistance *neo* gene into the *bciA* locus[1].

A PCR fragment of the *bchJ* locus amplified from the wild-type genome was about 1.8 kbp (Fig. S1D, lane 4), while those from the Δ*bchJ* (lane 5) and Δ*bciA*/*bchJ* (lane 6) mutants were about 2.6 kbp, i.e., 0.8-kbp longer by the insertion of the *aadA* gene in the *bchJ* locus. DNA sequences of the PCR fragments were determined to further confirm the mutation in the *bchJ* locus. The *bciA* loci were also confirmed using primers, sphaA-comf-F and sphaA-comf-R[1]. A PCR fragment from the Δ*bciA*/*bchJ* mutant (Fig. S1D, lane 3) was 1.3-kbp longer than those of the wild-type (lane 1) and Δ*bchJ* strains (lane 2) by the insertion of the *neo* gene into the *bciA* locus[1].

Reference in Supplementary text

1. Harada, J., Mizoguchi, T., Tsukatani, Y., Yokono, M., Tanaka, A., & Tamiaki, H. Chlorophyllide *a* oxidoreductase works as one of the divinyl reductases specifically involved in bacteriochlorophyll *a* biosynthesis. *J. Biol. Chem.* **289**, 12716–12726 (2014).

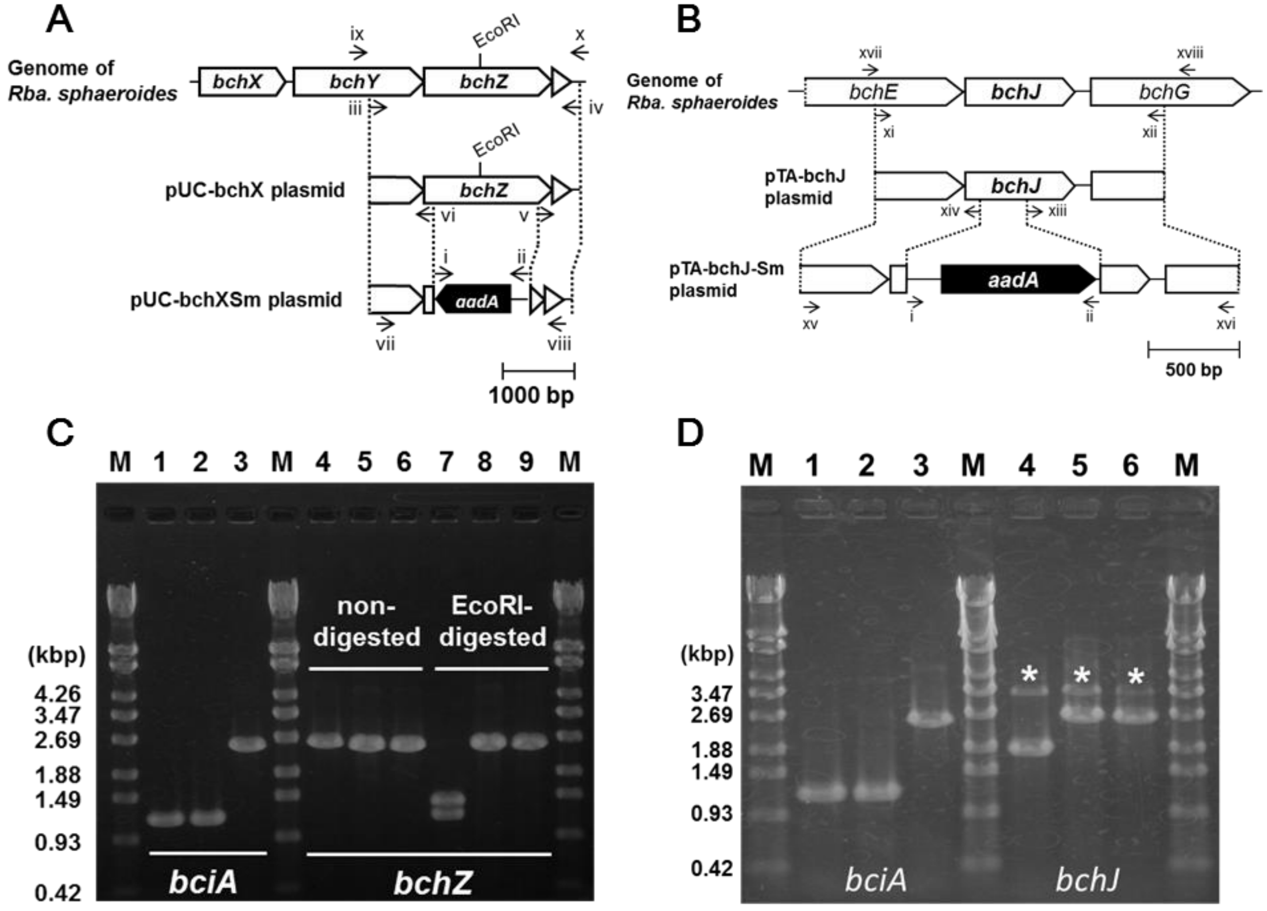

Figure S1. (A, B) Schematic map for the construction of the Δ*bchZ* and Δ*bchJ* mutants of *R. sphaeroides*, respectively. Genes are indicated by rectangles. The *aadA* gene confers resistance to streptomycin. Arrows represent the primers used for the mutant construction: i; aadA-F, ii; aadA-R, iii; bchZ-F, iv; bchZ-R, v; bchZ-inf-FI, vi; bchZ-inf-RI, vii; bchZ-inf-FII, viii; bchZ-inf-RII, ix; bchZ-comf-F, x; bchZ-comf-R, xi; bchJ-F, xii; bchJ-R, xiii; bchJ-inf-FI, xiv; bchJ-inf-RI, xv; bchJ-inf-FII, xvi; bchJ-inf-RII, xvii; bchJ-comf-F, xviii; bchJ-comf-R (see also Table S1 for each primer sequence and numbering). (C) PCR analyses using genomic DNAs extracted from the wild-type (lanes 1, 4, and 7), Δ*bchZ* (lanes 2, 5, and 8), and Δ*bciA*/*bchZ* strains (lanes 3, 6, and 9) of *R. sphaeroides*. Lanes 1-3 represent PCR products using primers, sphaA-comf-F and sphaA-comf-R[1], to amplify the *bciA* locus. Lanes 4-9 represent PCR products when using bchZ-comf-F and bchZ-comf-R primers to amplify the *bchZ* locus. (D) PCR analyses using genomic DNAs extracted from the wild-type (lanes 1 and 4), Δ*bchJ* (lanes 2 and 5), and Δ*bciA*/*bchJ* strains (lanes 3 and 6) of *R. sphaeroides*. Lanes 1-3 represent PCR products using primers, sphaA-comf-F and sphaA-comf-R[1], to amplify the *bciA* locus. Lanes 4-6 represent PCR products when using bchJ-comf-F and bchJ-comf-R primers to amplify the *bchJ* locus. The PCR bands shown with asterisks in lanes 4-6 are non-specifically amplified fragments. A DNA molecular size marker was loaded on lanes M, and the numbers indicate the lengths of the DNA marker fragments in kilobase(s).

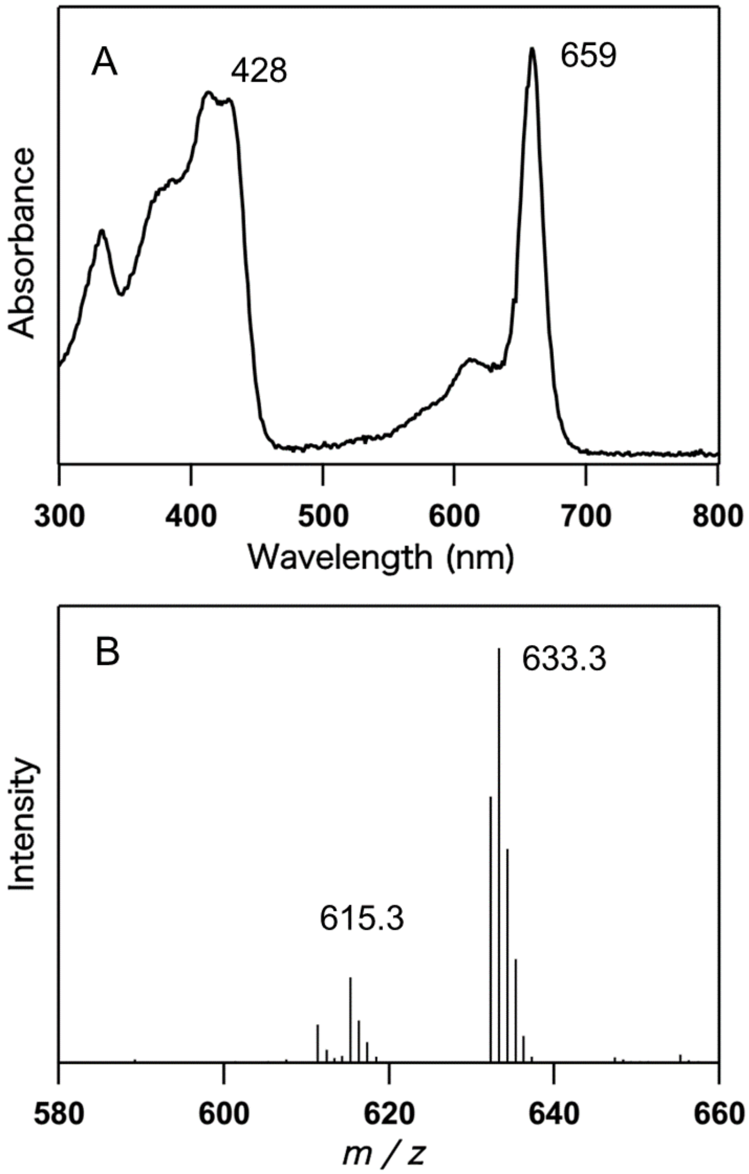

Figure S2. In-line (A) absorption and (B) mass spectra of the elution peak of a minor hydrophilic pigment component from the Δ*bchZ* mutant. The elution peak was observed at around 4 min in Fig. 2B, trace *i*. The elution time and the in-line absorption and mass spectra of the minor component are almost identical to those shown in Fig. 4B (peak 1) and Fig. 4CD (traces 1). Therefore, the minor hydrophilic pigment component of the Δ*bchZ* mutant would be assigned to 3-(1-hydroxyethyl)-Chlide *a*.

Table S1. Sequences of primers for construction of plasmids and for confirmation of mutants.

Primer name	Primer sequence	Primer number shown in Fig. S1	Overlapped sequence for In-Fusion reaction or restriction site (underlined)
aadA-F	CTGTTCGGTTCGTAAGCTGT	i	
aadA-R	CGTCGGCTTGAACGAATTGT	ii	
bchZ-F	ATGGCATCGCCGCCGACA	iii	
bchZ-R	TAAGACTGACGCCACATGCT	iv	
bchZ-inf-FI	TCGTTCAAGCCGACGCGTAGAGG-<br>AGCATCCGGTT	v	overlapped with aadA-R
bchZ-inf-RI	TTACGAACCGAACAGAATGCAGC-<br>ACCGAGGTCAC	vi	overlapped with aadA-F
bchZ-inf-FII	TCGAGCTCGGTACCCTATGAGGG-<br>CTCCGAGCTGA	vii	overlapped with MCS of pJSC vector
bchZ-inf-RII	CTCTAGAGGATCCCCACCATGCC-<br>CTCCCGATTAAT	viii	overlapped with MCS of pJSC vector
bchZ-comf-F	GGCGATCCATCCCTTCTAC	ix	
bchZ-comf-R	TATCAGCCATGCTATCCTCC	x	
bchJ-F	CCTCCTGGGCTGCCTCAA	xi	
bchJ-R	CGGCTCGACCGAATAGGC	xii	
bchJ-inf-FI	TCGTTCAAGCCGACGGCCCGC-<br>TGTCATTCGAGAT	xiii	overlapped with aadA-R
bchJ-inf-RI	TTACGAACCGAACAGTCCAGC-<br>ACCGGAATGAGCT	xiv	overlapped with aadA-F
bchJ-inf-FII	TCGAGCTCGGTACCCCCTCCTG-<br>GGCTGCCTCAA	xv	overlapped with MCS of pJSC vector
bchJ-inf-RII	CTCTAGAGGATCCCCCGGCTCG-<br>ACCGAATAGGC	xvi	overlapped with MCS of pJSC vector
bchJ-comf-F	GGTTCTACATGAAGAAGGCG	xvii	
bchJ-comf-R	TTGAGCGTCATGATGCCATG	xviii	
ppucf6	ATAGTCGACTTCACTGGGATTT-<br>TGCGCCC		SalI
pjr6	TATGGTACCGATATCAgagaccCCG-<br>CggtctcGGCGCCGCCCTTCTCGAA-<br>CTGCGGATGCGACC		BamHI, BsaI (boxed)

KOBsal-f1	TTCAGGCGCTCCCGAAGATCCCG-GGCCGTCTCTTGG	
KOBsal-r1	AAGAGACGGCCCGGGATCTTCGG-GAGCGC	
Spc2f1	ATAGAGCTCTAGATAATGCAAGT-AGCGTATGC	SacI
Spc2r1	ATAGAGCTCTAGAGCGGATGTTG-CGATTACTTCG	SacI
BvYZ-infu-F1	CGAGAAGGGCGGCGCCAGGGCT-GCCAGTTACGTTC	overlapped with BsaI site of pJN7 vector
BvYZ-infu-R1	CTGGGTACCGATATCTCACGCAG-CCTGCCCCCCGACA	overlapped with BsaI site of pJN7 vector
Gm-JN7-F	CTGCGTGAGATATCGCAACTGGT-CCAGAACCTTGA	overlapped with KpnI site pJ7-BvYZ
Gm-JN7-R	GGGAACAAAAGCTGGAAGCTTG-CATGCCTGCAGG	overlapped with KpnI site pJ7-BvYZ

MCS = multi-cloning site
